# Mast cells in a murine lung ischemia-reperfusion model of primary graft dysfunction

**DOI:** 10.1186/s12931-014-0095-0

**Published:** 2014-08-13

**Authors:** John R Greenland, Xiang Xu, David M Sayah, Feng Chun Liu, Kirk D Jones, Mark R Looney, George H Caughey

**Affiliations:** Medical Service, VA Medical Center, San Francisco, CA USA; Department of Medicine, University of California, San Francisco, CA USA; Department of Medicine, University of California, Los Angeles, CA USA; Department of Pathology, University of California, San Francisco, CA USA; Cardiovascular Research Institute, University of California, San Francisco, CA USA

## Abstract

Primary graft dysfunction (PGD), as characterized by pulmonary infiltrates and high oxygen requirements shortly after reperfusion, is the major cause of early morbidity and mortality after lung transplantation. Donor, recipient and allograft-handling factors are thought to contribute, although new insights regarding pathogenesis are needed to guide approaches to prevention and therapy. Mast cells have been implicated in ischemic tissue injury in other model systems and in allograft rejection, leading to the hypothesis that mast cell degranulation contributes to lung injury following reperfusion injury.

We tested this hypothesis in a mouse model of PGD involving reversible disruption of blood flow to one lung. Metrics of injury included albumin permeability, plasma extravasation, lung histopathology, and mast cell degranulation. Responses were assessed in wild-type (*Kit*^*+/+*^) and mast cell-deficient (*Kit*^*W-sh/W-sh*^) mice. Because mouse lungs have few mast cells compared with human lungs, we also tested responses in mice with lung mastocytosis generated by injecting bone marrow-derived cultured mast cells (BMCMC).

We found that ischemic lung responses of mast cell-deficient *Kit*^*W-sh/W-sh*^ mice did not differ from those of *Kit*^*+/+*^ mice, even after priming for injury using LPS. Degranulated mast cells were more abundant in ischemic than in non-ischemic BMCMC-injected *Kit*^*W-sh/W-sh*^ lungs. However, lung injury in BMCMC-injected *Kit*^*W-sh/W-sh*^ and *Kit*^*+/+*^ mice did not differ in globally mast cell-deficient, uninjected *Kit*^*W-sh/W-sh*^ mice or in wild-type *Kit*^*+/+*^ mice relatively deficient in lung mast cells.

These findings predict that mast cells, although activated in lungs injured by ischemia and reperfusion, are not necessary for the development of PGD.

**Electronic supplementary material:**

The online version of this article (doi:10.1186/s12931-014-0095-0) contains supplementary material, which is available to authorized users.

## Introduction

Although lung transplantation treats otherwise incurable lung diseases, it carries a 5-year mortality of nearly 50%. Reperfusion injury, also known as primary graft dysfunction (PGD), is defined clinically by radiographic lung opacities consistent with edema and by high requirements for supplemental O_2_ during the first 72 hours of reperfusion [1]. PGD affects up to 25% of transplanted lungs and is the major cause of early morbidity and mortality after transplantation. Allograft recipients surviving severe PGD are more likely to be physiologically impaired one year after transplantation and to be more vulnerable to consequences of acute rejection. Moreover, they are more likely to develop bronchiolitis obliterans syndrome (BOS), a manifestation of chronic rejection [2]. Overall, PGD is a major barrier to success of lung transplantation, and new insights regarding pathogenesis are needed to guide approaches to prevention and therapy [3-5].

Mast cells have been implicated in the pathogenesis of several types of ischemia-reperfusion injury. In mouse models of ischemia-reperfusion injury to muscle, the extent of tissue damage correlates with mast cell degranulation and is markedly reduced in mice lacking mast cells. Release of mouse mast cell protease-5, an elastolytic protease related to human mast cell chymase, appeared to be critical the development of reperfusion injury in skeletal muscle [6]. Mast cell-deficient mice also have a less severe phenotype after ischemia-reperfusion injury to myocardium [7]. Mast cell stabilizers and anti-histamines protect against myocardial ischemia-reperfusion injury [8].

Mast cells abound at baseline in donor lung airway walls and alveolar interstitia. Their numbers may increase following transplantation and in association with acute rejection and BOS [9,10]. Furthermore, mRNAs encoding mast cell-specific products, such as tryptase, are abundant in transbronchial biopsies of human allografts [11]. Studies in animals suggest that lung mast cells also can be activated in the setting of ischemia-reperfusion. For example, in rat tracheal allografts, mast cells degranulate and upregulate chemokine ligand expression [12], and in dog lungs, mast cells appear to be recruited and to degranulate following transient ligation of a pulmonary artery [13]. Traditional mast cell stabilizers, such as ketotifen and sodium cromoglycate, decrease inflammation following lung reperfusion in rats, as evidenced by decreased levels of ICAM-1 and TNFα and increased NOS-2 [14,15].

There are mechanistic reasons as well to suspect a role for mast cells in PGD. Mast cell products, especially secreted TNFα and proteases (such as tryptases, which are the major secreted proteins of human mast cells), promote neutrophilic inflammation, which is a hallmark of PGD [16-19]. Also, mast cells express adenosine receptors and are activated by adenosine [20-22], which accumulates in ischemic tissue prior to re-establishment of perfusion as a by-product of ATP utilization and depletion.

One of the challenges in using mice to model roles of mast cells in human lung pathology is that the numbers and distribution of mast cells differ between laboratory mice and humans. A traditional way to explore the contributions of mast cells to pathology in mice is to compare phenotypes in wild-type mice with those in one of several available strains of mice lacking mast cells due to genetic defects in expression of c-Kit. If differences are seen, then greater certainty about mast cell involvement can be obtained by restoring the wild-type phenotype via adoptive transfer of wild-type bone marrow-derived cultured mast cells (BMCMC). However, the lung is challenging in this regard. While *Kit*^*W-sh/W-sh*^ mice lack lung mast cells in all sites, wild-type *Kit*^*+/+*^ C57BL/6 mouse lung contain mast cells primarily in the trachea and peribronchial tissues with very few in lung parenchyma. However, parenchymal mast cells can be observed following intravenous injection of BMCMC into mast cell-deficient *Kit*^*W-sh/W-sh*^ mice in a C57BL/6 background [23-25]. The density of the mast cell population in the alveolar interstitium of *Kit*^*W-sh/W-sh*^ mice following intravenous injection of BMCMC is substantially higher than in wild-type *Kit*^*+/+*^ mice [23,26,27]. Interestingly, in humans, alveolar interstitia support perhaps the highest density of mast cells in any normal tissue [28]. Therefore, these results of adoptive transfer of BMCMC into *Kit*^*W-sh/W-sh*^ mice suggested the possibility of “humanizing” mouse lungs with respect to alveolar mast cell density by injecting mice with BMCMC. As a basis for conducting the present study, we hypothesized that mast cell activation and degranulation contribute to the pathogenesis of PGD in lung allografts. We found that mast cells are present and that they degranulate in human PGD and in a mouse model of PGD with alveolar mastocytosis. However, we did not detect differences in the degree of lung injury related to the presence of mast cells in mice, suggesting that they do not make major contributions to ischemia-perfusion injury in lung tissues.

## Materials and methods

Studies on human tissue were performed with written consent from the subject and with approval of the UCSF Human Research Protection Program Committee on Human Research (Protocol #13-10738). All non-human animal studies were approved by the UCSF IACUC in accordance with the NIH Guide for the Care and Use of Laboratory Animals.

### Histology

Human lung biopsy tissue sections were stained with hematoxylin and eosin or with c-kit antibody (A4502, Dako, Carpinteria CA). Mouse tissue was fixed in 4% paraformaldehyde and specimens were embedded in paraffin and cut into 5-μm sections and stained with hematoxylin and eosin. To identify mast cells in mice and humans, sections were deparaffinized and stained with 0.5% acidified toluidine blue.

### Mouse ischemia reperfusion model

Mast cell–deficient C57BL/6 Kit^W-sh/W-sh^ (Wsh) and wild-type C57BL/6 *Kit*^*+/+*^ (+/+) mice were housed as described [23]. For some experiments, 0.1 mg/kg of LPS (Sigma-Aldrich, St. Louis MO) was injected intraperitoneally 24 hours before surgery. Experiments were performed at 8-12 weeks of age with the exception of adoptive transfer experiments, which were performed at 20-22 weeks to allow mast cell populations to establish and mature in the lung. Mice were anesthetized with ketamine and xylazine, and 0.5 mL of PBS was administered intraperitoneally. The trachea was intubated, and animals were mechanically ventilated (MiniVent, Harvard Apparatus, Holliston MA) with 100% oxygen, respiratory rate of 120/min, tidal volume of 10 ml/kg and positive end-expiratory pressure of 3 cm of H_2_O. Anesthesia was maintained with 1% vaporized isoflurane. After left thoracotomy, the left pulmonary hilum was isolated and circumscribed with a suture. Heparin (0.03 U/g of mouse weight) was administered intraperitoneally and the hilar suture was tied using a slipknot to occlude the hilar structures, or left untied in sham surgery. The tidal volume was reduced to 8 ml/kg and the respiratory rate increased to 150/min. The thoracotomy was closed with sutures and the hilar slipknot suture tunneled outside of the chest. Buprenorphine analgesia was administered, the animal was extubated after awakening from anesthesia, and was allowed to recover in a warmed chamber with supplemental O_2_ administered at 2 L/min. One hour after hilar occlusion, ^125^I-labelled albumin was administered intraperitoneally, the slipknot suture was removed, and the animal was transferred to a warmed cage. Four hours after reperfusion, animals were euthanized by anesthetic overdose prior to blood and lung collection. Lungs were homogenized in 1 ml of water. Extravascular lung water was determined by the gravimetric methods and endothelial permeability to ^125^I-labeled albumin was used to calculate extravascular plasma equivalents using the equations detailed in the Additional file 1 [29].

### Mast cell adoptive transfer

BMCMC were derived by culturing bone marrow from C57BL/6 mice for 5-6 weeks in 10 ng/ml of IL-3, as previously described [23]. Stem cell factor (50 ng/ml) was added to the cultures starting at weeks 3. BMCMC (10^7^ in 0.2 ml of PBS) were injected via tail vein into 5-week old mice. Ischemia-reperfusion experiments were performed at least 12 weeks after adoptive transfer.

### Statistical analysis

Differences between groups were assessed using Students t-test or one-way ANOVA with Bonferroni-corrected post-test comparisons between pre-specified groups using Prism version 5.0a (GraphPad Software, Inc., La Jolla, CA). Power calculations were performed using R (version 3.0.2, R Foundation for Statistical Computing, Vienna, Austria).

## Results

### Degranulated mast cells in human lung with PGD

**Figure 1:**
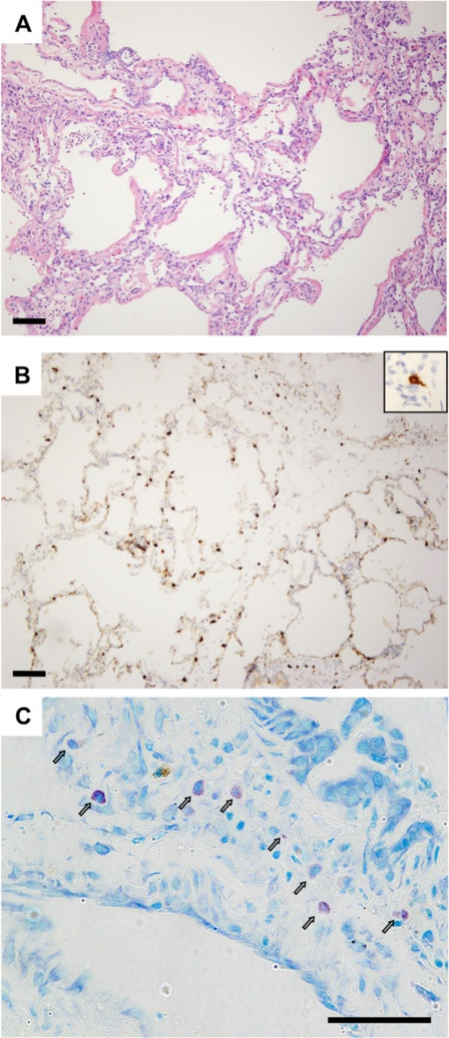
**Mast cells in human lung in setting of primary graft dysfunction. (A)** Hematoxylin and eosin staining in a lung biopsy taken 3 days following transplant in a patient with severe PGD. **(B)** Mast cells were identified by immunostaining for c-Kit (brown cells). A higher-magnification example of a degranulated mast cell is shown in the inset. **(C)** Toluidine blue staining shows mast cells (open arrows) in a variety of stages of degranulation as indicated by irregular borders and heterogeneously reduced staining. Scale bars: 100 μm in **(A)** and **(B)** and 50 μm in **(C)**.

Considering multiple lines of evidence supporting potential roles of mast cells in potentiating PGD, we obtained tissue from a patient who had had a tissue biopsy done at the time of PGD to evaluate the frequency and degranulation state of mast cells. In this case, the patient had a right lower lobe lung biopsy on post-operative day 3 performed at the time of transition from veno-arterial to veno-venous extracorporeal membrane oxygenation following lung transplantation. Histological examination of biopsy sections showed diffuse alveolar septal thickening with edema, type II pneumocyte hyperplasia, hyaline membranes, and airspace neutrophilic infiltrates, consistent with primary graft failure (Figure 1A). A C4d stain was negative. Staining with antibodies specific for c-Kit (Figure 1B) demonstrated mast cells distributed throughout the areas of injury. Per high-powered field (0.196 mm^2^), there were 22 ± 7 mast cells. We determined that 33% of mast cells identified by c-kit staining had irregular borders consistent with degranulation. Toluidine blue staining (Figure 1C) demonstrated an even greater frequency of degranulation, with 59% of mast cells having heterogeneously reduced staining. However, toluidine blue staining may underestimate the total number of mast cells in formalin-fixed samples [30,31]. A previous study found 14 mast cells per high power field in normal lungs, 18-32 during acute rejection, and 39 in BOS [9].

### Mouse model of ischemia-reperfusion acute lung injury

**Figure 2:**
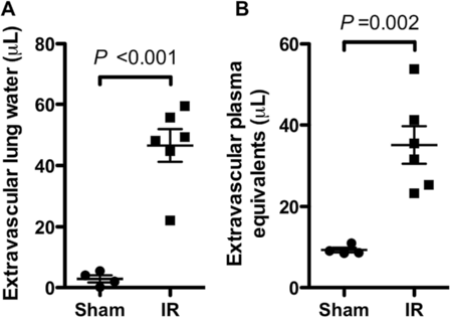
**Dysfunction in mouse lungs subjected to ischemia-reperfusion (IR) versus in sham-operated lungs.** In these experiments, blood flow to the left lungs of wild-type (*Kit* ^*+/+*^) C57BL/6 mice was interrupted by hilar ligation for 1 h followed by 4 h of reperfusion, then lung harvest (n = 6). Control mice underwent sham ligation (n = 4). Lung water **(A)** and extravascular plasma equivalents **(B)** were determined in the ligated and sham-ligated lungs as parameters of lung injury.

The presence of degranulated mast cells in lung tissue from a patient with PGD motivated the development of a mouse model to test whether mast cell degranulation contributed to ischemia-reperfusion injury. Ischemia was induced using 1 hour of left hilar ligation followed by 4 hours of reperfusion. Compared to animals undergoing thoracotomy and sham ligation with placement of an untied suture, the hilum-ligated animals had significantly increased levels of extravascular lung water and endothelial permeability to albumin (extravascular plasma equivalents) in the left lung (Figure 2). Using these results, we determined that with 5 animals per group this model would have 80% power to detect a greater than or equal to 48% change in extravascular plasma equivalents and a 54% change in extravascular lung water in the absence of mast cells by two-sided *t*-test with a type I error probability (alpha level) of 0.05.

### Lung injury following ischemia-reperfusion injury in mast cell-deficient mice

**Figure 3:**
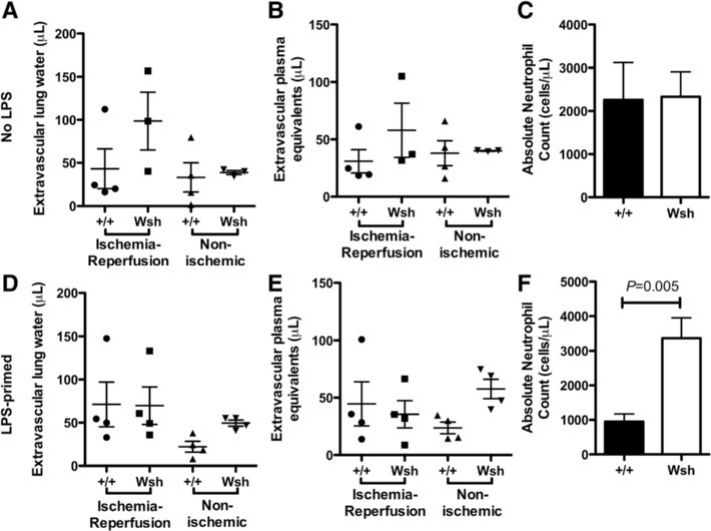
**Lung function after ischemia-reperfusion injury in wild-type** ***Kit*** ^***+/+***^ **mice versus mast cell-deficient** ***Kit*** ^***W-sh/W-sh***^ **mice without (A-C) and with (D-F) LPS priming. (A, D)** Lung water and **(B, E)** extravascular plasma equivalents were compared in left lungs, which were subjected to ischemia-reperfusion, and in right lungs, which were unligated controls. Four mice were analyzed per group except in the *Kit* ^*W-sh/W-sh*^ without LPS group, in which one animal did not survive surgery. The bar graphs **(C, F)** show results of peripheral blood neutrophil concentrations determined in samples obtained at the time of lung harvest.

To test the hypothesis that mast cells contribute to worsened injury following ischemia reperfusion, we compared injury using this model in mast cell–deficient *Kit*^*W-sh/W-sh*^ and wild-type *Kit*^*+/+*^ mice. As shown in Figure 3A-B, we did not observe differences in extravascular lung water (*P* = 0.22) or extravascular plasma equivalents (*P* = 0.56) between the two mouse strains in the left (ipsilateral) or right (contralateral) lung.

One critique of the *Kit*^*W-sh/W-sh*^ model is that mast cell deficiency is accompanied by hematopoeitic abnormalities, including neutrophilia [32]. As increased neutrophil infiltration could worsen lung injury, potentially counteracting a possible decrease in injury resulting from absent mast cells, we compared neutrophil counts between the two strains following ischemia reperfusion injury (Figure 3C). No difference in peripheral blood neutrophil counts was observed between the two strains (*P* = 0.95).

Because lungs of mice raised in specific pathogen-free conditions are not exposed to the same environmental stimuli as transplanted lungs in humans, it has been suggested that the mouse immune system may be relatively under-primed. Indeed, we have previously shown that mice housed in a specific pathogen-free barrier facility require LPS priming to achieve levels of acute lung injury similar to those seen in non-barrier mice [33]. Accordingly, we hypothesized that pre-treatment with LPS might amplify a possible difference in injury between *Kit*^*W-sh/W-sh*^ and wild-type *Kit*^*+/+*^ animals.

As shown in Figure 3D-E, we did not observe a significant difference between wild-type and *Kit*^*W-sh/W-sh*^ animals with respect to the degree of ischemia-reperfusion injury following pre-treatment with LPS. There was no difference between extravascular lung water (*P* = 0.21) or extravascular plasma equivalents (*P* = 0.28) by ANOVA. Overall, levels of injury were comparable between the LPS treated and untreated groups. Interestingly, we did observe an increase (*P* = 0.005) in the frequency of peripheral blood neutrophils in LPS-treated mice (Figure 3F). We did observe a trend towards increasing extravascular plasma equivalents in contralateral lungs of the LPS-primed *Kit*^*W-sh/W-sh*^ animals relative to wild-type controls (*P* = 0.10), which may be a consequence of this systemic neutrophilia.

### Adoptive transfer of mast cells results in a “humanized” murine lung parenchyma

**Figure 4:**
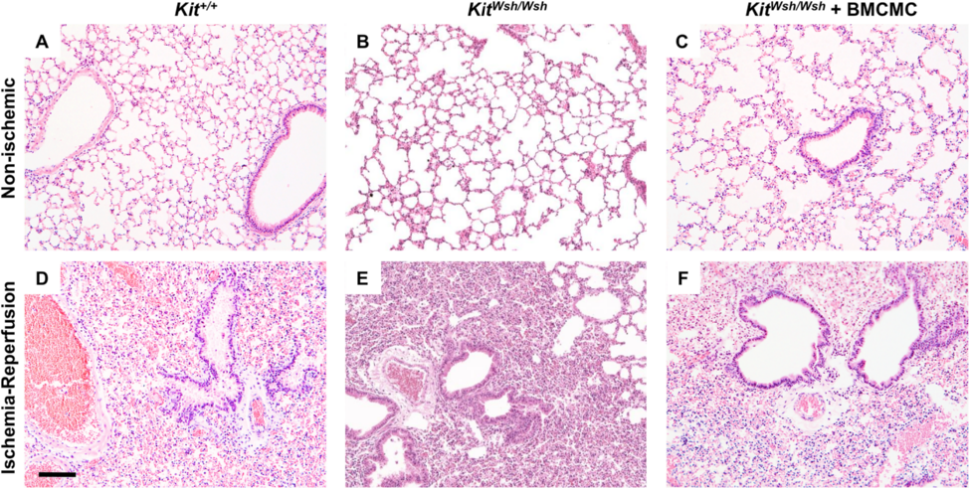
**Assessment of lung histopathology in mice following ischemia-reperfusion injury.** Pulmonary mastocytosis was generated by injection BMCMC into *Kit* ^*W-sh/W-sh*^ mice tail veins and waiting 18 weeks to allow maturation. Hematoxylin and eosin staining in left lungs subjected to ischemia-reperfusion injury **(D-F)** as compared with contralateral non-ischemic **(A-C)** lungs demonstrates lung injury in the wild-type *Kit* ^*+/+*^ **(A, D)**, mast cell deficient *Kit* ^*W-sh/Wsh*^ **(B, E)**, and BMCMC-injected *Kit* ^*W-sh/Wsh*^ mice **(C, F)**. Scale bar denotes 100 μm.

Having confirmed the previously reported funding [27] that mouse lung parenchyma has few mast cells compared with human lung parenchyma, we evaluated ischemia-reperfusion injury in a potentially more relevant mouse model. Following adoptive transfer of mast cells by the intravenous route, mast cells repopulate the lung parenchyma over a period of 4-12 weeks [23]. We assessed ischemia-reperfusion injury in animals following injection of BMCMC. As shown in Figure 4, lung tissue from wild-type, *Kit*^*W-sh/W-sh*^, and BMCMC-injected *Kit*^*W-sh/W-sh*^ animals subjected to ischemia-reperfusion demonstrated alveolar septal thickening, edema, and hyaline membrane deposition, consistent with the pattern of injury seen in human tissue with PGD. By contrast, the contralateral lungs demonstrated minimal injury.

**Figure 5:**
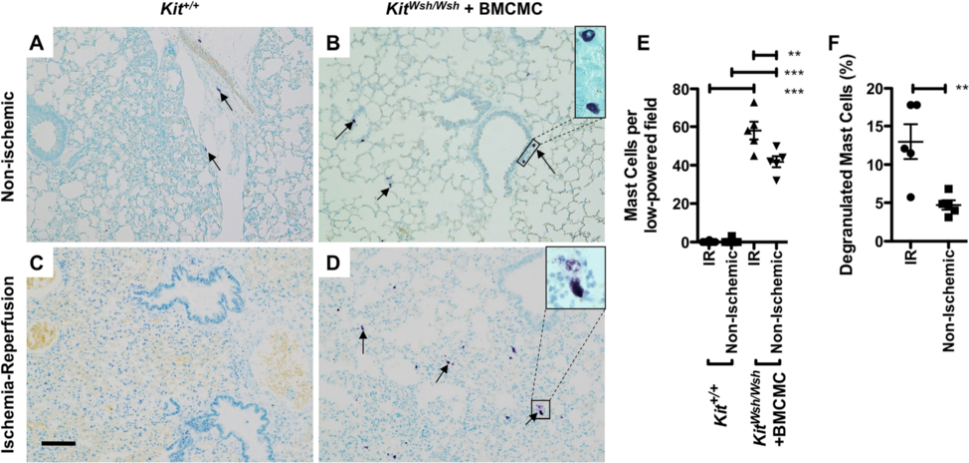
**Comparison of lung histopathology and density of intact and degranulated mast cells in mice following ischemia-reperfusion injury.** Toluidine blue staining is shown for left lungs subjected to ischemia-reperfusion **(C-D)** and contralateral non-ischemic lungs **(A-B)** after ischemia-reperfusion injury. Degranulation was detected by the finding of metachromatic cells with indistinct, granulated borders in tissue sections stained with toluidine blue. Examples of parenchymal mast cells are shown by arrows and are enlarged in the insets. Scale bar denotes 100 μm. **(E)** In random low-power microscopic fields, mean counts of metachromatic toluidine blue-staining cells were compared in lungs subjected to ischemia-reperfusion injury and from contralateral control lungs from *Kit* ^*W-sh/W-sh*^ mice with mastocytosis generated by injection of BMCMC and from untreated wild-type animals (n = 5 per group). The percentage of degranulated mast cells is compared in BMCMC-injected *Kit* ^*W-sh/W-sh*^ mice between ischemia-reperfusion and contralateral non-ischemic lungs **(F)**. **P < 0.01; ***P < 0.001.

We assessed mast cells in lungs from animals following adoptive transfer of BMCMC by histology (Figure 5A-D). Injection of BMCMC led to a significant increase in lung parenchymal mast cell density. Following ischemia-reperfusion, as shown in Figure 5E, we observed 58 ± 10 mast cells per low powered field (LPF) in ipsilateral lungs from BMCMC-treated *Kit*^*W-sh/W-sh*^ animals, compared with 42 ± 6 mast cells in contralateral lungs from BMCMC-treated *Kit*^*W-sh/W-sh*^ animals, <1 mast cell in ipsilateral and contralateral lungs in wild-type animals (*P* <0.001 by ANOVA). There was an increased density of mast cells in the ipsilateral lungs of BMCMC-treated *Kit*^*W-sh/W-sh*^ animals both when compared with contralateral lungs (*P* <0.001) and when compared with wild-type animals not treated with BMCMC (*P* <0.001). As seen in Figure 5F, we found increased frequency of degranulated mast cells following ischemia reperfusion injury in ipsilateral (13 ± 5%) as compared with contralateral lungs (5 ± 1%, *P* <0.01). Together, these findings show that mast cells are present and that they degranulate in this model, consistent with findings in human PGD.

### Lung injury following ischemia-reperfusion in a mouse with alveolar mastocytosis

**Figure 6:**
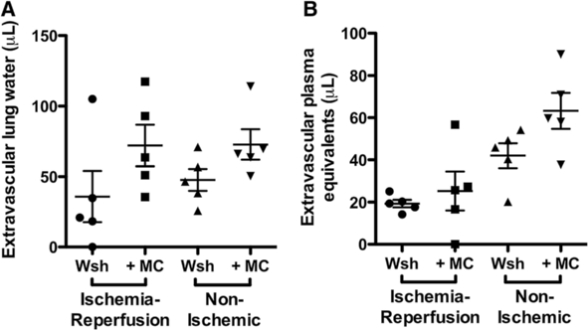
**Comparison of lung responses to ischemia-reperfusion injury in mice with and without parenchymal mastocytosis induced in** ***Kit*** ^***W-sh/W-sh***^ **by injection with BMCMC (n = 5 per group).** Left lungs were subjected to ischemia-reperfusion, while right lungs were unligated controls. Adoptive transfer of mast cells did not change the quantity of **(A)** lung water in the ischemic (*P* = 0.09) or contralateral (*P* = 0.23) lungs, nor did mast cells affect the extravascular plasma equivalents **(B)** in ischemic (*P* = 0.56) or contralateral (*P* = 0.06) lungs.

**Figure 7:**
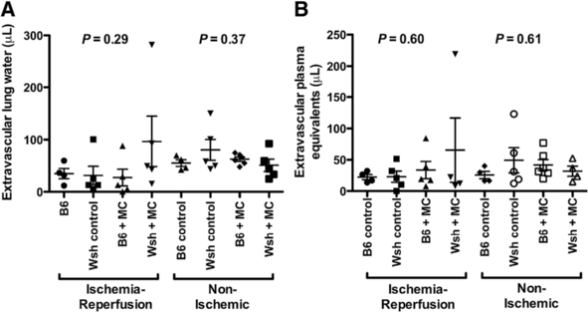
**Comparison of lung responses to ischemia-reperfusion injury in C57BL/6** ***Kit*** ^***+/+***^ **(B6) and** ***Kit*** ^***W-sh/W-sh***^ **(Wsh) mice with and without parenchymal mastocytosis induced by injection with BMCMC (n = 5 per group, except that one B6 control animal did not survive surgery).** Lung water **(A)** and extravascular plasma equivalents **(B)** were compared in ischemic left lungs following reperfusion and in unligated right lungs. *P*-values for a one-way ANOVA test are shown for each metric and side.

To assess the role of mast cells in mediating ischemia reperfusion injury in this adoptive transfer model, we compared measurements of lung injury between *Kit*^*W-sh/W-sh*^ mice with and without reconstitution with BMCMC (Figure 6). There were no significant differences in extravascular plasma equivalents or extravascular lung water between the with- and without-BMCMC groups (*P* <0.05). To exclude the role of the *Kit*^*W-sh/W-sh*^ background as a cause for no observed difference between the reconstituted and untreated animals, we performed adoptive transfer of mast cells in both *Kit*^*W-sh/W-sh*^ and *Kit*^*+/+*^ wild-type animals. As shown in Figure 7, we detected no differences between the mastocytosis treated animals and the untreated animals in either lung following ischemia-reperfusion as assessed by extravascular plasma equivalents or extravascular lung water.

## Discussion

Motivated by the observation of degranulated mast cells in tissue obtained from a patient with PGD, we developed a mouse model of ischemia-reperfusion injury with variation of mast cell density to evaluate the potential role of mast cells in mediating PGD. This model recapitulated the histologic hallmarks and mast cell degranulation and increased mast cell density observed in human pathology. However, we did not observe a significant effect of mast cells across multiple permutations of this experimental model.

Although we did not observe mast cell-dependent effects on lung extravascular water or endothelial permeability, it is likely that ischemia reperfusion injury results in mast cell-dependent effects on other parameters that were not tested. For example, pharmacologic inhibition of mast cells during reperfusion injury has been shown to decrease ICAM-1 and increase cGMP and NOS-2 levels following reperfusion injury [14]. Further, mast cells are distinct from other cell types in that secondary activation is required for upregulation of Hypoxia-Ischemia Factor Iα in response to ischemia [34] and hypoxia results in autocrine secretion of IL-6, which promotes mast cell survival [35]. The unique responses of mast cells to ischemia suggest that they may yet play a role in ischemia reperfusion injury through mechanisms that could not be observed in this model. We selected ischemia and reperfusion times to achieve a relatively severe and reproducible level of injury, but it is possible that varying ischemia and reperfusion times might bring out more subtle differences related to mast cell degranulation.

Prior studies had shown a role for mast cell degranulation in mediating cardiac and skeletal muscle ischemia-reperfusion injury [6,8] and had shown that mast cells are present and active during ischemia-reperfusion injury in tracheal and lung tissue [12,13]. The present data suggest, however, that despite being active, mast cells do not play a non-redundant role in mediating reperfusion injury to the lung. Multiple independent mechanisms are thought to contribute PGD, including oxidative stress, calcium and iron overload, hypercoaguability, cell adhesion molecule upregulation, pro-inflammatory cytokines, membrane lipid remodeling, complement activation, endothelin release, and activation of leukocytes, macrophages, lymphocytes, and neutrophils. Mast cells may contribute to some, but not all of these mechanisms [36].

Beyond effects related to ischemia-reperfusion injury, studies in mice have suggested that mast cells may help to determine the fates of transplanted organs by effects on immune tolerance [37]. For example, studies of skin allografts suggested that mast cells promote tolerance [38], because mast cell-deficient mice could not tolerate allografts. This effect may be due to depression of IL-6 levels in the allograft by the mast cell tryptase mMCP-6 [39], which can cleave and inactivate IL-6 [19]. On the other hand, studies in *Kit*^*W-sh/W-sh*^ mice undergoing experimental cardiac transplantation showed no significant differences in rejection when compared to wild-type animals as manifested by graft inflammatory cells, cytokine or adhesion molecule expression, or coronary artery disease [40]. Thus, it is possible that for adaptive immune-mediated rejection, the contribution of mast cells to allograft dysfunction may also be redundant.

This study has limitations. For example, a small effect from mast cell degranulation might not be evident without large study populations, especially given the variability intrinsic to the utilized measures of lung injury. A meaningful effect would likely have been evident in at least one of the described experimental approaches. It is also possible that mast cell degranulation has an important role in ischemia-reperfusion injury but that other cell types can compensate for the absence of mast cells. The lack of observed difference in wild-type animals with and without mast cells, however, suggests that mast cell degranulation beyond what is physiologic in mice does not lead to worsened lung injury. Although we do not have reason to suspect that adoptively transferred mast cells would lose their functionality, we cannot exclude the possibility that the reconstituted mast cells have some dysfunction despite preserved ability to degranulate. Finally, the present model employed warm ischemia, which is physiologically distinct from the cold ischemia present during lung transplantation because of the metabolic changes induced by hypothermia. Similar levels of ischemia-reperfusion injury have been reported independent of hypothermia [41] and mast cell activation has been reported to be unlinked to hypothermia [42].

In summary, we found that mast cell frequency did not alter the severity of ischemia-reperfusion injury in mouse lungs. These findings suggest that strategies targeting mast cells for the prevention of PGD may not be effective when used independent of other injury pathways.

## Additional file

Additional file 1:
**Equations used in the calculation of mouse lung injury parameters.**
