# Supplementary material for: Mast cells in a murine lung ischemia-reperfusion model of primary graft dysfunction
Source: Respir Res. 2014 Aug 13;15(1):95. doi: 10.1186/s12931-014-0095-0 (PMC4151192; doi:10.1186/s12931-014-0095-0)
Supplement: Additional file 1: — Equations used in the calculation of mouse lung injury parameters. [file 12931_2014_95_MOESM1_ESM.pdf]

Supplemental Information

Equations used to determine mouse lung injury:

$$Excess\ lung\ water\ (ELW) = Wt_{lung+added\ water} * F_{wh} - Wt_{blood\ in\ lung} * F_{wb} - Wt_{added\ water} - 0.051\text{g}$$

$$Extravascular\ plasma\ equivalents\ (EVPE) = \frac{C_{lung-blood}}{C_{plasma} * 1000}$$

$$C_{lung-blood} = C_{lung} - \left(Q_b * (1 - Hct) * C_{plasma}\right)$$

$$C_{plasma} = \frac{C_{blood}}{Wt_{blood} * (1 - Hct)}$$

$$Blood\ volume\ in\ lung\ (Q_b) = \frac{1.039 * Wt_{lung+added\ water} * F_{wh} * Hg_{supernatant}}{F_{ws} * Hg_{plasma}}$$

$$Fraction\ water\ in\ homogenate\ (F_{wh}) = \frac{Wt_{wet\ lung} - Wt_{dry\ lung}}{Wt_{wet\ lung}}$$

$$Fraction\ water\ in\ blood\ (F_{wb}) = \frac{Wt_{wet\ blood\ sample} - Wt_{dry\ blood\ sample}}{Wt_{wet\ blood\ sample}}$$

$$Fraction\ water\ in\ supernatant\ (F_{ws}) = \frac{Wt_{wet\ supernatant} - Wt_{dry\ supernatant}}{Wt_{wet\ supernatant}}$$

C= counts from ^{125}I-labeled Iodine

Wt = weight
